# What Do You Believe In? French Translation of the FAD-Plus to Assess Beliefs in Free Will and Determinism and Their Relationship with Religious Practices and Personality Traits

**DOI:** 10.5334/pb.321

**Published:** 2017-02-20

**Authors:** E. A. Caspar, O. Verdin, D. Rigoni, A. Cleeremans, O. Klein

**Affiliations:** 1 Université libre de Bruxelles (ULB), Consciousness, Cognition and Computation Group (CO3), Center for Research in Cognition & Neurosciences (CRCN), ULB Neuroscience Institute (UNI), BE; 2 Université libre de Bruxelles (ULB), Center for Social and Cultural Psychology (CSCP), BE; 3 Université libre de Bruxelles (ULB), BE; 4 Ghent University, Department of Experimental Psychology, BE

**Keywords:** Free will, Determinism, Religion, FAD-plus

## Abstract

The influence of (dis)belief in free will on prosocial behaviors and sense of control has attracted considerable interest over the last few years. The provision of relevant research tools to assess beliefs in free will and determinism for the community thus becomes a central endeavour. However, no relevant validated questionnaires are currently available to the French language community. Therefore, the present study was aimed at providing a valid French translation of the FAD-plus (Paulhus & Carey, 2011), a questionnaire built to assess people’s beliefs in Free will and Determinism. Exploratory factor analysis of the data obtained in Sample 1 revealed a four factor model. Confirmatory factor analyses on the basis of Sample 2 data were conducted to compare the theoretical model advanced by Paulhus and Carey’s versus the model obtained in Sample 1. With only but a few modifications as compared to the original questionnaire, the questionnaire that we here propose appears to constitute a reliable tool for the French language community. We also examined the relationship between beliefs in free will, determinism and religious practices. We found that the more people are engaged in religious practices, the more they believe in determinism and in the inevitability of their future.

## Introduction

Whether our life should be viewed as depending on our own free will or taken to be determined by forces out of our control is an ancient and fundamental question. Consider the World Cup quarterfinal match with England, when Diego Maradona scored a goal with the help of what he called the “Hand of God”. Was this goal truly the determination of God’s will, or was it a mere manifestation of Maradona’s skill in the context of the movements of the other players? Determinism holds that each event has anterior causes that could not have resulted in a different outcome than the actual outcome they produced. Believing in determinism has substantial implications about the manner in which we act. For instance, viewing oneself as a mere puppet does not square well with attempting to contest the existing social order. Such beliefs likewise have implications on how we judge the behaviour of other people: Moral praise and moral blame both presuppose free will.

These philosophical stances have long been considered as being completely incompatible with each other (Kane, 1999; Pereboom, 2001; Strawson, 1986). However, their relationship is rather complex (Stroessner & Green, 1990): Not only is it possible to believe in either one (i.e., the incompatibilist view) or in both (i.e., the compatibilist view), but there is also an entire array of possible intermediate positions (e.g., libertarianism, semicompatabilism, impossibilism, mysterianism, and so on). A great variety of arguments have been developed by philosophers to defend their position on free will (e.g. see Doyle, 2011 for a review of these positions). When motivating their theoretical position, philosophers often claim that it fits folk intuitions, or that it is commonsensical (Nahmias, Morris, Nadelhoffer & Turner, 2005). However, such beliefs about free will and related constructs have been largely neglected within the philosophical debate on free will. As pointed out by Wegner (2002), “*philosophers have given us plenty of «isms» to use in describing the positions that can be taken on this question, meanwhile not really answering it in a satisfying way*” (page *ix*). Nowadays, several experimental studies have investigated the extent to which beliefs in free will or in determinism influence different aspects of people’s decisions, such as for instance their moral behavior (Vohs & Schooler, 2008), their prosocial behavior and aggressiveness (Baumeister, Masicampo & DeWall, 2009), their job and academic achievement (Stillman et al., 2010; Feldman, Chandrashekar, & Wong, 2016), as well as the basic neural processes that subtend action control (Rigoni, Kühn, Sartori, & Brass, 2011; Rigoni, Pourtois, & Brass, 2015).

Paulhus and Margesson (1994) developed a new instrument — the Free will And Determinism scale (FAD-4) — to investigate beliefs in Free Will and in three related constructs, Scientific Causation (genes’ influence on behavior), Fate (people’s futures are determined in advance) and Chance. They were guided by the observation that the different scales available at that time all failed to adequately capture the multifaceted relation between free will and determinism (Keller, 2005; Rakos, Laurene, Skala & Slane, 2008; Viney, Waldman & Barchilon, 1982). Nevertheless, the FAD-4 has never been published as such, due to several limitations. First, the balance between pro-trait items and con-trait items was not respected. Only 5 items (out of 28) were reversals, and they exhibited double loadings and even cross-loadings with other factors. Second, subscale reliability sometimes slipped below .60. As a result, in 2011, Paulhus and Carey created the “Free will And Determinism Plus scale” (FAD-plus), a 27-items questionnaire that overcame some of the limitations of the FAD-4. First, only pro-traits items were included in this new questionnaire, but acquiescence, a bias frequently reported in questionnaires in which respondents tend to agree with all the questions, was controlled to avoid a bias in the positive direction. Second, they avoided philosophical jargon, making the questionnaire accessible to a wider population. Third, they used items that fit in only one subscale, hence avoiding cross-loadings between factors. In its final form, the instrument includes four factors (see Table 1): Free Will, Scientific Determinism, Fatalistic Determinism and Unpredictability. Paulhus and Carey (1994) made a distinction between scientific and fatalistic determinism. The first refers to scientific causality (i.e. “medicine can predict consequences of invisible and fatal bacteria on health and cure it”), whereas fatalism refers to inevitability. Participants used a 5-point Likert scale from 1 (Strongly disagree) to 5 (Strongly agree). Reliability of the FAD-plus (as measured by Cronbach’s Alpha) was satisfactory for all subscales. Paulhus and Carey (2011) reported a value of .70 for the Free Will subscale, of .69 for Scientific Determinism, of .82 for Fatalistic Determinism and of .72 for Unpredictability. A Confirmatory Factor Analysis (CFA) confirmed this four-factor structure.

**Table 1:** English and French versions of the FAD-plus.

	*English Version*	*French Version*

	**Free Will**	**Libre arbitre**

4	People have complete control over the decisions they make	Les gens ont un contrôle complet sur les décisions qu’ils prennent
8	People must take full responsibility for any bad choices they make	Les gens doivent endosser la pleine responsabilité des mauvais choix qu’ils ont fait
12	People can overcome any obstacles if they truly want to	Les gens peuvent surmonter tous les obstacles s’ils en ont vraiment envie
16	Criminals are totally responsible for the bad things they do	Les criminels sont totalement responsables des mauvaises actions qu’ils ont faites
21	People have complete free will	Les gens disposent d’un libre-arbitre complet
23	People are always at fault for their bad behavior	Les gens sont toujours en tort pour leur mauvais comportement
26	Strength of mind can always overcome the body’s desire	La force de l’esprit peut toujours surmonter les désirs du corps

	**Scientific Determinism**	**Déterminisme Scientifique**

2	People’s biological makeup determines their talents and personality	La constitution biologique des personnes détermine leurs talents et leur personnalité
6	Psychologists and psychiatrists will eventually figure out all human behavior	Les psychologues et les psychiatres finiront par comprendre tout le comportement humain
10	Your genes determine your future	Tes gènes déterminent ton avenir
14	Science has shown how your past environment created your current intelligence and personality	La science a montré comment ton environnement passé a créé ton intelligence et ta personnalité
18	As with other animals, human behavior always follows the laws of nature	Comme pour les autres animaux, le comportement humain suit toujours les lois de la nature
22	Parent’s character will determine the character of their children	Le caractère des parents détermine celui de leur enfant
24	Childhood environment will determine your success as an adult	L’environnement que tu as eu pendant ton enfance détermine ton succès en tant qu’adulte

	**Fatalistic Determinism**	**Déterminisme Fataliste**

1	I believe that the future has already been determine by fate	Je crois que l’avenir est déterminé par le sort
5	No matter how hard you try, you can’t change your destiny	Quelques soient les efforts que vous faites, vous ne pouvez pas changer votre destin
9	Fate already has a plan for everyone	Le destin est déjà planifié pour chacun
13	Whatever will be, will be-there’s not much you can do about it	Ce qui doit arriver arrivera, il n’y a pas grande chose que tu puisses faire
17	Whether people like it or not, mysterious forces seem move their lives	Que les gens aiment cela ou pas, des forces mystérieuses influencent leurs vies

	**Unpredictability**	**Imprédictibilité**

3	Chance events seem to be the major cause of human history	Les évènements dus au hasard semblent être la cause majeure de l’histoire de l’humanité
7	No one can predict what will happen in this world	Personne ne peut prédire ce qui va arriver dans ce monde
11	Life seems unpredictable-just like throwing dice or flipping a coin	La vie semble imprévisible, comme lancer un dé ou jouer à pile ou face
15	People are unpredictable	Les gens sont imprévisibles
19	Life is hard to predict because it is almost totally random	La vie est difficile à prédire car elle est presque entièrement aléatoire
20	Luck plays a big role in people’s lives	La chance joue un rôle important dans la vie des personnes
25	What happens to people is a matter of chance	Ce qui arrive aux gens est une question de hasard
27	People’s future cannot be predicted	L’avenir des gens ne peut pas être prédit

In this context, the main goal of the current study was to translate and validate the FAD-plus in French language in order to facilitate future research in francophone countries. To do so, we performed an Exploratory Factor Analysis (EFA) on a first sample and a CFA on a second sample. In addition, we assessed the construct validity of the best fitting model t by correlating the different subscales of the FAD-plus with the Big Five Inventory, a questionnaire measuring different aspects of personality (John & Srivasta, 1999) and with several religiosity scales assessing individuals’ religious practices (Saroglou, 2011).

In the literature, several studies have shown relationships between belief in free will and personality traits. For instance, Stillman and Baumeister (2010) suggested a relation between prosocial behaviour, such as agreeableness, and belief in Free will. Also, Paulhus and Carrey (2011) observed that Fatalistic determinism was positively correlated with agreeableness and negatively correlated with emotional stability. We therefore expected to replicate similar correlations to determine the construct validity of the French translation of the FAD-plus. In the same vein, the putative relationship between belief in Free will and religious practices has already been discussed in the literature. For instance, Caricati (2007) found that non-practicing believers obtained higher scores on a scale measuring belief in genetic determinism. Carey and Paulhus (2013) explored the relation between belief in free will versus determinism and religiosity by independently evaluating intrinsic and extrinsic religiosity. Individuals with intrinsic religiosity sincerely consider that religion is a part of their life and use their religion as a guide in all aspects of their life (Donahue, 1985; Kahoe, 1974), while individuals with an extrinsic orientation towards religiosity use religion as an end (Batson, 1982), for instance to establish or maintain a social network. In their study, Carey and Paulhus (2013) reported that belief in free will was positively correlated with religiosity, but only with intrinsic religiosity. In addition, they found that Fatalistic Determinism and Scientific Determinism were associated with extrinsic religiosity. Therefore, we used scales that assess religious practices in order to validate our French validation of the FAD-plus. The scale that we used measures four aspects of religious practices: Believing (the connection between human spirit and a spiritual entity), Bonding (the emotional dimension of religiosity), Behaving (the norms of the religion) and Belonging (the need for a social identity). Saroglou (2011) suggested that Believing and Behaving are both forms of intrinsic religiosity. Therefore, we expected a positive correlation between these two subscales and the Free Will subscale of the FAD-plus.

## Method

**Samples and Procedure.** The questionnaire has been translated by using the back-translation procedure. The questionnaire was initially translated in French by a native French speaker and then translated back to English by a native English speaker. Finally, the first author of the original questionnaire compared the initial and final versions. The translation was fine-tuned to reduce discrepancies. Table 1 reports our translation organized following the dimensions identified by Paulhus and Carey (2011).

To validate the French translation and the structure of the FAD-plus, we used two samples. Sample 1 included 997 participants who were contacted via social networks. The majority of respondents were employees and the remaining participants were undergraduates in psychology. The study was approved by the ethical committee of the Université libre de Bruxelles and participants gave their consent after reading the information about the questionnaires. Age and sex were recorded. Of these 997 questionnaires, 286 were removed due to incomplete data. Incomplete data were not taken into account because they were mainly composed of the personal information about the participants without further answers to the questionnaires. Of the 711 remaining questionnaires, the majority had been completed by female participants (N = 442). The mean age was 26.47 years old (SD = 8.752; range: 18–74). The data obtained in Sample 1 were analysed with an Exploratory Factor Analysis (EFA).

Sample 2 included 291 participants, who anonymously replied to an online survey posted on several student university networks in Belgium’ French speaking community. Age, sex, nationality, native language and educational level were recorded. Of these 291 questionnaires, 96 were removed due to incomplete data. On the 195 remaining questionnaires, the majority had been completed by female participants (N = 138) and the mean age was 25.72 years old (SD = 8.48; range: 17–66). The data obtained in Sample 2 were analysed with Confirmatory Factor Analyses (CFA). All participants in the two samples completed the FAD-plus. Participants in Sample 2 also completed the French version of the Big Five Inventory questionnaire and questionnaires assessing their religiosity.

**Instruments. ***FAD-plus*: The FAD-plus (Paulhus & Carey, 2011) measures belief in free will using 27 items rated on a 5-point scale, ranging from “1” (Strongly agree) to “5” (Strongly disagree). Items were distributed in four factors: Free Will, Scientific Determinism, Fatalistic Determinism and Unpredictability.

*Big Five Inventory*: the Big Five Inventory (John & Srivasta, 1999 – for the French translation, see Plaisant, Courtois, Réveillère, Mendelsohn & John, 2010) is widely used in the scientific community. It measures the five most important factors of personality, namely: Extraversion, Agreeableness, Conscientiousness, Neuroticism and Openness. It includes 44 items rated on a 5-point scale, ranging from “1” (Strongly disagree) to “5” (Strongly agree). The “Openness” factor refers to people’s degree of curiosity and creativity. “Conscientiousness” refers to the tendency to be organized, aiming for achievement, and showing self-discipline. “Extraversion” refers to outgoing and energetic personalities. “Agreeableness” is characterized by friendly and compassionate behaviour and “Neuroticism” is the tendency to experience unpleasant emotions, such as anxiety and depression.

*Religiousness Scales*: we used several scales to measure religiosity. The first one, the Big Four Religious Dimensions and Cultural Variation, measured four dimensions in religiosity: Believing, Bonding, Behaving and Belonging (see Saroglou, 2011). It includes 12 items rated on a 7-point scale, ranging from “1” (Not at all) to “5” (Totally). Independent items measuring the degree of religiosity of participants composed the second scale (Saroglou & Galand, 2004; Saroglou & Munoz-Garcia, 2008). The first three items asked, using a 5-point scale, ranging from “1” (Not at all) to “5” (Very important), how much God, religion and spirituality are important. The fourth item asked participants, on a Scale from “1” (Not at all) to “5” (A lot, almost every days), how frequently they pray, independently of official ceremonies.

## Results and analysis

We conducted a Principal Component Analysis (PCA) on the relationships among the FAD-plus items with direct oblimin rotation and used this structure as a baseline model which we analysed with confirmatory factor analysis (CFA) in AMOS 21, comparing it with the original model obtained by Paulhus and Carey (2011).

**Descriptive Statistics.** Table 2 reports the means and standard deviations of the four subscales for each gender in the two samples. In Sample 1, we found three significant gender differences. Men obtained higher scores on Scientific Determinism (2.88, SD = .51) than women (2.74, SD = .55), *t*(709) = –3.427, *p* = .001, d = –0.26 (see Table 2). Women scored higher on both Fatalistic Determinism (2.07, SD = .73) and Unpredictability (3.18, SD = .59) than men (1.85, SD = .66 and 3.08, SD = .61), respectively *t*(709) = 3.960, *p* < .001, d = 0.31, and t(709) = 2.243, *p* = .025, d = 0.17. In Sample 2, we found that women exhibited marginally higher scores on Free Will (3.26, SD = .69) than men (3.02, SD = .80), *t*(193) = 2.109, *p* = .036, d = 0.33. In addition, we found, in Sample 1, a negative correlation between Unpredictability and age (*r* = –.165, *p* < .001). In Sample 2, we observed a positive correlation between Free will and age (*r* = .135, *p* = .051). We did not find any differences as a function of educational level in Sample 2.

**Table 2:** Means scores across gender.

	*Sample 1*				*Sample 2*			

	Males		Females		Males		Females	

	*Mean*	*SD*	*Mean*	*SD*	*Mean*	*SD*	*Mean*	*SD*

**FW**	3.21	.61	3.16	.60	3.05	.77	3.26	.65
**SD**	2.88	.51	2.74	.55	2.86	.51	2.73	.62
**FD**	1.85	.66	2.07	.73	1.95	.86	1.94	.71
**UN**	3.08	.61	3.18	.59	3.01	.66	3.11	.61

Note: FW = Free Will; SD = Scientific Determinism; FD = Fatalistic Determinism; UN = Unpredictability.

**Exploratory factor analysis.** An Exploratory Factor Analysis (EFA) with direct oblimin rotation was conducted on the Sample 1 data. Bartlett’s test of sphericity was significant (< .001), which indicates inter-correlations among items. The KMO (Kaiser-Meyer-Olkin) value was .775, which indicates a reliable sample size for factor analysis. We chose to select four factors for extraction, based on Paulhus and Carey. We supressed small coefficients below 0.25. The four emerging factors explained 38.45% of the variance. Globally, the factorial structure was similar to the structure observed by Paulhus and Carey (2011). The first factor explained 13.35% of the variance and was composed of the items of the Fatalistic Determinism subscale. The second factor explained 10.15% of the variance, and corresponded to the Free will subscale. The third factor explained 8.46% of the variance and corresponded to the Unpredictability subscale. The fourth factor explained 6.49% of the variance and consisted of items from the Scientific Determinism subscale. Cronbach’s alpha was .69 for free will, .58 for scientific determinism, .73 for fatalistic determinism and .71 for unpredictability. We found that item 27 (“People’s futures cannot be predicted”) loaded on two scales, showing a coefficient of -.527 on Unpredictability and -.376 on Fatalistic determinism. In addition, item 6 (“Psychologists and psychiatrists will eventually figure out human behavior”) did not load on any of the four factors (see Table 3 for the factor loadings). We thus decided to remove these two items.

**Table 3:** Pattern matrix from the exploratory factor analysis in Sample 1.

	Fatalistic Determinism	Free Will	Unpredictability	ScientificDeterminism

9. Fate already has a plan for everyone	**.80**	.10	.05	–.04
5. No matter how hard you try, you can’t change your destiny	**.72**	–.06	.04	.06
13. Whatever will be, will be-there’s not much you can do about it	**.64**	.03	–.14	.02
1. I believe that the future has already been determine by fate	**.60**	–.11	–.11	.10
17. Whether people like it or not, mysterious forces seem move their lives	**.60**	.06	–.04	–.10
16. Criminals are totally responsible for the bad things they do	.04	**.65**	–.01	.03
21. People have complete free will	–.07	**.64**	.00	–.06
12. People can overcome any obstacles if they truly want to	.04	**.60**	–.04	–.14
23. People are always at fault for their bad behavior	.03	**.59**	–.01	.10
4. People have complete control over the decisions they make	–.07	**.58**	.11	.02
8. People must take full responsibility for any bad choices they make	–.06	**.56**	.00	.13
26. Strength of mind can always overcome the body’s desire	.07	**.45**	–.05	.00
19. Life is hard to predict because it is almost totally random	.04	.08	**–.72**	–.05
11. Life seems unpredictable-just like throwing dice or flipping a coin	.12	–.02	**–.70**	–.04
25. What happens to people is a matter of chance	.15	–.05	**–.60**	.09
3. Chance events seem to be the major cause of human history	.02	–.15	**–.53**	.20
27. People’s future cannot be predicted	**–.38**	–.00	**–.53**	–.06
7. No one can predict what will happen in this world	–.09	.12	**–.51**	–.05
20. Luck plays a big role in people’s lives	.13	–.11	**–.49**	.18
15. People are unpredictable	.15	.22	**–.44**	–.18
2. People’s biological makeup determines their talents and personality	.07	–.12	.05	**.70**
10. Your genes determine your future	.20	–.05	.20	**.60**
22. Parent’s character will determine the character of their children	–.06	0.7	–.08	**.60**
24. Childhood environment will determine your success as an adult	–.01	–.04	–.09	**.57**
14. Science has shown how your past environment created your current intelligence and personality	–.13	.18	–.18	**.49**
18. As with other animals, human behavior always follows the laws of nature	.09	.24	–.07	**.26**
6. Psychologists and psychiatrists will eventually figure out all human behavior	.01	.16	.09	.24

**Confirmatory factor analysis.** We conducted a CFA to confirm the exploratory model on the Sample 2 data. The CFA method proposed a chi-squared (χ^2^) value for which the null hypothesis means that all observed parameters correspond to the theoretical model. A good model fit provides a non-significant result at the 0.05 threshold (Barrett, 2007). To propose a validation of the model, we reported several classical fit indices (Byrne, 2001): CMIN/df, AGFI, CFI and RMSEA. CMIN/df is an index to consider when the Chi squared reached significance, because it adjusts the Chi squared according to the sample size and degrees of freedom. It must be below 3 (Kline, 1998). The Adjusted Goodness of Fit (AGFI) measures the proportion of variance/covariance explained by the model, compared to the absence of model. Following Hu and Bentler (1995), AGFI is acceptable from .90 and above (the maximum is 1). The Comparative Fit Index (CFI) is an index of comparison between the observed model and an independent model. Several indices are available to measure this comparison, but the CFI seems to be the best indicator, because it also corrects sensibility for little samples. Bentler (1990) indicated that the CFI is acceptable above .90. The final index that we computed is the Root Mean Square Error of Approximation (RMSEA). It measures the approximate error of the model by degree of freedom and it is “good” when it falls between .05 and .08 and “very good” when inferior to .05. Because RMSEA is sensitive to sample size, Confidence Intervals (CI) proposed at 90% and the p of Close Fit (Pclose) are reported. A Pclose greater than .5 suggests that the model is close to the fit (Jöreskog & Sörbom, 1996). In addition to those fit indices, assessing the statistical significance of parameter estimates through the Critical Ratio (CR) can help to consider if items are relevant for the model or not (Byrne, 2001). The CR represents the parameter estimates divided by its standard error. If a parameter is not significant, the item is not relevant for the model.

We conducted a CFA on both the four factors obtained by Paulhus and Carey (2011) and the four adjusted factors that we had obtained based on the EFA. Then, we compared the two models with their respective Akaike Information Criterion (AIC). The best model is generally the one with the smallest AIC value.

**Figure 1:**
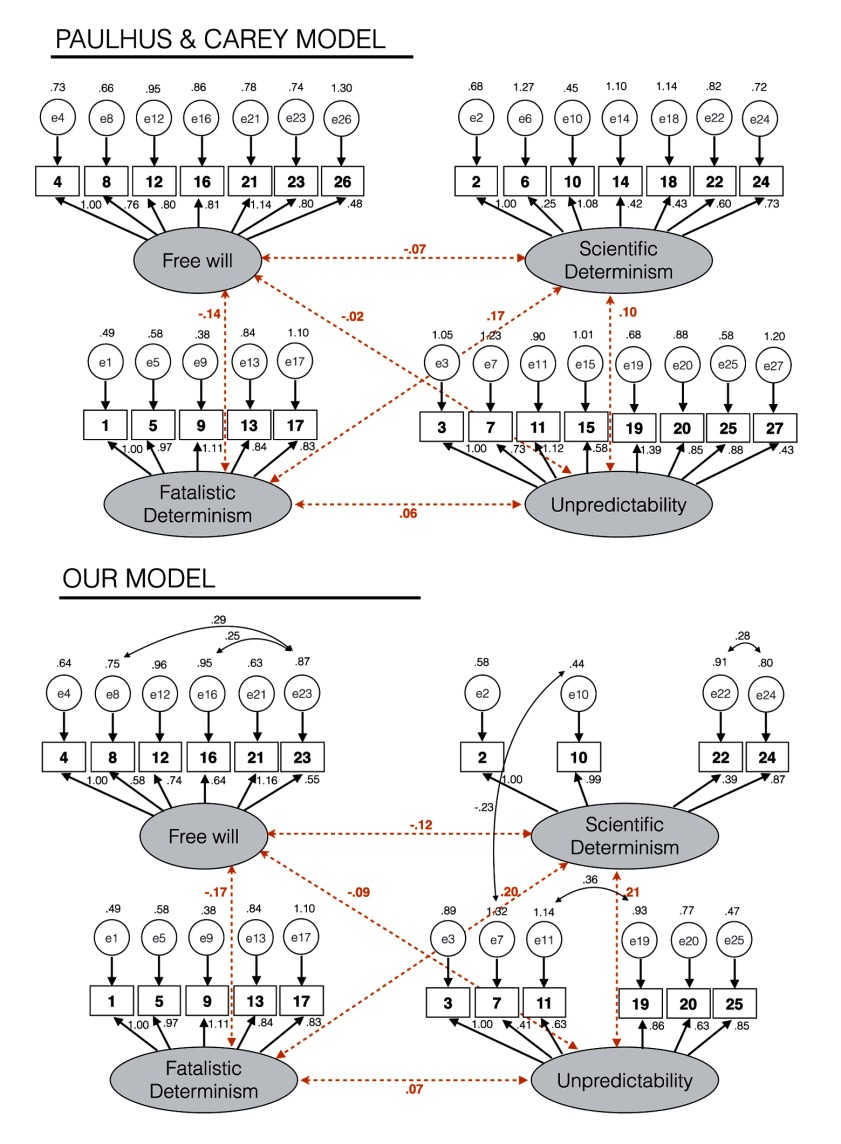
On the top, graphical representation of the model of Paulhus & Carey (2011) and on the bottom, graphical representation of our final model for the Frensh Translation.

**Paulhus and Carey’s model.** The original model by Paulhus and Carey showed mediocre fit, and only a few fit indices were acceptable. Chi squared reached significance – χ^2^_(318)_ = 575.78; *p* < .001, but the CMIN/df correction was under the recommended threshold of 3 (1.811), suggesting that the significance of the effect was due to the sample size. All structural parameters were significant, except Item 6 (*p* < .07). It is worth noting that items 14, 18, 26 and 27 were significant, but their Critical Ratio (CR) scores were smaller than for other items. For the different values of indices, we obtained: AGFI = .784, CFI = .755, RMSEA = .065 (90% CI = low: .056 – high: .073) with a Pclose of .003. AIC had the value of 749.78 (Figure 1). This confirms that the theoretical model of Paulhus & Carey (2011) does not fit well with the actual French translation of the FAD-plus. The main reason is probably because their model includes items 6 and 27, which we found to be unreliable.

**Our model.** We then performed a CFA on our model, without items 6 and 27, based on the EFA results. Overall, even if AIC criterion was smaller than in the Paulhus and Carey’s model (653.69) thus indicating that this model is more parsimonious than the model of Paulhus and Carey, fit indices were not better (see Model 1, Table 4). Chi squared reached significance – χ^2^_(269)_ = 412.115; *p* < .001, but the CMIN/df correction was under the recommended threshold of 3 (1.828). For the different values of indices, we obtained: AGFI = .795, CFI = .779, RMSEA = .065 (90% CI = low: .056 – high: .074) with a Pclose of .004. We thus tried to identify the reasons of these low scores. We found that CR scores were smaller for items 14, 15, 18 and 26 than for the other items (item 14: 3.053; item 15: 3.421; item 18: 3.164; item 26: 3.480). In addition, the coefficients for these items across the different subscales were also smaller than .320 (item 14 for Scientific Determinism: .260; item 15 for Unpredictability: .311; item 18 for Scientific Determinism: .270; item 26 for Free will: .293). We thus decided to remove these items for the next CFA. Half of the fit indices were acceptable (see Model 2, Table 4). Chi squared reached significance – χ^2^_(203)_ = 290.449; *p* < .001, but the CMIN/df correction was under the recommended threshold of 3 (1.823). For the different values of indices, we obtained: AGFI = .807, CFI = .819, RMSEA = .065 (90% CI = low: .054 – high: .076) with a Pclose of .011. Indices were better than those previously obtained but not sufficient to propose a reliable model. We thus tried again to identify the reasons. We observed that Modification Indices indicated that a covariation between e22 and e24, between e16 and e23, between e8 and e23, between e11 and e19 and between e9 and e25 would improve the model fit. We decided to replicate the analysis allowing covariances between these errors. Overall, we obtained satisfactory fit indices. Chi squared reached significance – χ^2^_(203)_ = 249.33; *p* < .001, but the CMIN/df correction was under the recommended threshold of 3 (1.401). For the different values of indices, we obtained the following: AGFI = .860, CFI = .920, RMSEA = .045 (90% CI = low: .037 – high: .058) with a Pclose of .707. This model was satisfactory (see Model 3, Table 4). In addition, AIC was 355.33 which is the smallest value across the different models, indicating that this model is the most parsimonious.

**Table 4:** Fit Indices Sample 1 and Sample 2.

*Fit Indices*				

	Sample 1 Paulhus & Carey (2011)’s Model	Sample 2 Model 1	Sample 2 Model 2	Sample 2 Model 3

χ^**2**^	575.78	412.115	290.449	249.33
**df**	318	269	203	178
**CMIN/df**	1.811	1.828	1.823	**1.401**
**AGFI**	.784	.795	.807	.860
**CFI**	.755	.779	.819	**.920**
**RMSEA**	.065	.065	.065	**.045**
**90% IC**	.056 – .073	.056 – .074	.054–.076	**.037–.058**
**Pclose**	.003	.004	.011	**.707**
**AIC**	749.78	653.69	469.99	**355.33**

Note: χ^2^ = Chi squared; df = degrees of freedom; CMIN/df = minimum discrepancy/degrees of freedom; AGFI = Adjusted Goodness of Fit; CFI = Comparative Fit Index; RMSEA = Root Mean Square Error of Approximation; 90% IC = RMSEA 90% Interval Confidence. Values in Bold reach acceptability, following literature.

**Construct validity.** We investigated the construct validity of the subscales of our final model by correlating them with the Big Five Inventory and religiosity scales (Believing, Binding, Behaving and Belonging). The Fatalistic Determinism subscale was positively correlated with the four dimensions of the religiousness scale (Believing: *r* = .368, *p* < .001; Binding: *r* = .307, *p* < .001; Behaving: *r* = .319, *p* < .001; Belonging: *r* = .345, *p* < .001). None of the other subscales were correlated with the religiousness scale. In addition, the Fatalistic determinism subscale was positively correlated with independent items measuring the degree of religiosity of participants (see Sarouglou & Galand, 2004; Saroglou & Munoz-Garcia, 2008). People for who God is important in their life, for whom religion is important for their life and for who spirituality is important for their life believe more in Fatalistic Determinism (r = .347, *p* < .001; *r* = .326, *p* < .001; *r* = .391, *p* < .001, respectively). The Free will subscale was negatively correlated with the importance of spirituality in their lives (*r* = –.157, *p* < .03). None of the other factors were correlated with these items.

Concerning the Big Five Inventory, we found a positive correlation between Free will and Conscientiousness (*r* = .228, *p* = .001) and a negative correlation between Free will and Neuroticism (*r* = .160; *p* < .03). Inversely, Fatalistic determinism were positively correlated with Neuroticism (*r* = .157, *p* < .03). No significant correlations were found with Unpredictability or Scientific Determinism.

**Orthogonality of factors.** In our final model, we found a significant negative correlation between Free will and Fatalistic determinism (*r* = –.215, *p* = .003). This correlation suggests that the more people believe in free will, the less they tend to believe in fatalism. Additionally, we found positive correlations between Unpredictability and Scientific Determinism (*r* = .216, *p* = .002), suggesting that the more people believe in Unpredictability, the more they tend to believe that science could predict the future. Fatalistic Determinism and Scientific Determinism were also positively correlated (*r* = .232, *p* = .001), revealing that people who believe in one form of determinism also tend to believe that this also is true for other forms of determinism.

## Discussion

In this study, we wanted to provide a valid French translation of the FAD-plus. We thus conducted a CFA based on the four-factor model described by Paulhus and Carey (2011), including Free Will, Scientific Determinism, Fatalistic Determinism and Unpredictability. The final version is presented in the Appendix.

The CFA conducted on Paulhus and Carey’s model was not entirely satisfactory. Most of the fit indices did not reach acceptable thresholds. Therefore, we looked at the possible sources of misspecification. We found several misspecifications, which was suggestive of a high degree of overlap in item content. This overlap between errors was present only between items within the same subscales. It is possible that these items, although worded differently, ask the same question within the same subscale. In addition, we removed six items based on the EFA (Sample 1) and on the subsequent CFA (Sample 2). One possibility would be to re-test the questionnaires with a different translation for these items. However, even without these items, the final model reached the acceptability threshold for all the fit indices, suggesting that our final questionnaire is a valid translation of the FAD-plus in French language.

The construct validity showed several expected links between the different subscales of the FAD-plus and other questionnaires. Specifically, the Fatalistic determinism subscale was positively correlated with the four dimensions of the religiosity scale and with independent items measuring the degree of religiosity of participants. This suggests that the higher people are engaged in religious practices, the more they believe in fatalistic determinism and the inevitability of their future. This is consistent with the widespread view that people engaged in religious practices believe that their destiny depends on God’s will (Baumeister, Bauer, & Lloyd, 2010).

The positive correlation between Conscientiousness and Free will was not surprising, because conscientious individuals are assumed to control their impulses and thus can develop a higher sense of control on their daily actions (Lynn et al., 2013; Rigoni et al., 2011). Finally, we observed that Fatalistic determinism was positively correlated with Neuroticism. Belief in fatalism implies that people believe they lack control over future events affecting their lives. It is reasonable to assume that a lack of control over one’s own life is associated with the neurotic spectrum (e.g. anxiety, frustration, and so forth). This pattern of correlations confirms previous evidence that, believing to be in control of one’s own behavior is associated with positive outcomes and is ultimately beneficial for one’s psychological well-being (e.g. Taylor & Brown, 1988).

Paulhus and Carey (2011) found a link between Unpredictability and Fatalistic Determinism in all their three studies. They argued that these two beliefs have unknowability and unpredictability in common, such as a need for mystery. Interestingly, we found the same relation between these subscales, thus confirming this assumption. We also observed that the relation between Free will and Fatalistic Determinism was negative, which attune well with the incompatibilist perspective. According to Nichols (2004), most people adopt an incompatibilist position: they think that they have free will and that they are responsible for their actions, which are thus not experienced as pre-determined. However, the incompatibilist viewpoint has not always been identified in prior studies (Nahmias et al., 2005, 2006; Nichols & Knobe, 2007). For instance, Nahmias et al. (2005, 2006) tested folk judgment of free will and moral responsibility across several studies. They found that the majority of participants, even in a deterministic scenario, judge the agent as having free will and as being responsible for his acts. They claimed that most people would endorse a compatibilist position. In addition, judgments of responsibility are highly context-sensitive. When participants read a specific scenario in which emotions are involved, their vision tends to espouse compatibilism. Nichols and Knobe (2007) presented participants with a scenario in which they had to “*imagine that in the next century we discover all the laws of nature, and we build a supercomputer which can deduce from these laws of nature and from the current state of everything in the world exactly what will be happening in the world at any future time*” (pp.667). Authors showed that, even in a scenario judged by participants to be deterministic, people are nevertheless judged to be responsible for their actions if their actions are bad or immoral. Thus, an inverse relationship between these two subscales could also have been observed. However, most scenarios used in previous studies (Nahmias et al., 2005, 2006; Nichols & Knobe, 2007) pointed to scientific determinism (e.g. “Your genes determine your future”) rather than to fatalistic or religious determinism. It is therefore possible that people can intuitively reconcile free will with scientific determinism, but not with fatalistic determinism. In addition, it should be noted that in the current study the questionnaire was not presented in the context of a scenario, which could explain why we found an incompatibilist view between these two subscales.

A limitation of the current study concerns the size of Sample 2. Even if all parameter indices were significant, thus suggesting that the sample size was sufficient, some researchers consider that a sample of more or less 200 participants is fair, but it should reach 500 or even more to be very good or excellent (MacCallum, Widaman, Zhang & Hong, 1999).

To conclude, we have provided a valid French translation of the FAD-plus scale. Importantly, we highly recommend avoiding using the six items that we have removed, because the model fit was superior without them. There is an increasing number of studies that investigate the impact of (dis)belief in free will on prosocial behaviors (e.g. Alquist, Ainsworth, & Baumeister, 2013; Bamfield & Horton, 2009; Baumeister, Masicampo & DeWall, 2009; Krueger, Hoffman, Walter, & Grafman, 2014; Leotti, Iyengar, & Ochsner, 2010; Shariff et al., 2014; Stillman, et al., 2010; Vohs & Schooler, 2008; Wegner, 2002) and sense of control (Aarts & van den Bos, 2011; Lynn, Dessel, & Brass, 2013; Lynn, Muhle-Karbe, Aarts & Brass, 2014; Rigoni, Kühn, Sartori, & Brass, 2011), either by means of questionnaires assessing participants’ beliefs in free will and determinism, or by means of induced beliefs. Therefore, offering reliable tools to promote further research in this burgeoning domain is particularly important. We hope our adaptation of the FAD-plus will prove to be such a tool.

## Additional Files

The additional files for this article can be found as follows:

10.5334/pb.321.s1

DOI: https://doi.org/10.5334/pb.321.s1

## References

[1] Aarts H., van den Bos K. (2011). On the foundations of beliefs in free will intentional binding and unconscious priming in self-agency. Psychological Science.

[2] Alquist J. L., Ainsworth S. E., Baumeister R. F. (2013). Determined to conform: Disbelief in free will increases conformity. Journal of Experimental Social Psychology.

[3] Bamfield L., Horton T. (2009). Understanding attitudes to tackling economic inequality.

[4] Baumeister R. F., Bauer I. M., Lloyd S. A. (2010). Choice, free will, and religion. Psychology of Religion and Spirituality.

[5] Baumeister R. F., Masicampo E. J., DeWall C. N. (2009). Prosocial benefits of feeling free: Disbelief in free will increases aggression and reduces helpfulness. Personality and Social Psychology Bulletin.

[6] Bentler P. M. (1990). Comparative Fit Indexes in Structural Models. Psychological Bulletin.

[7] Byrne B. M. (2001). Structural equation modeling with AMOS: Basic concepts, applications and programming.

[8] Caricati L. (2007). Power of genetics: Adaptation and validation of a scale for measuring belief in genetic determinism (BGD) with classical test analysis and Rasch analysis. TPM–Testing, Psychometrics, Methodology in Applied Psychology.

[9] Doyle B. (2011). Free Will: *The scandal in Philosophy*.

[10] Feldman G., Chandrashekar S. P., Wong K. F. E. (2016). The freedom to excel: Belief in free will predicts better academic performance. Personality and Individual Differences.

[11] Hu L.-T., Bentler P. M., Hoyle, Rick H. (1995). Evaluating model fit.

[12] John O. P., Srivastava S., Pervin L. A., John O. P. (1999). The Big-Five trait taxonomy: History, measurement, and theoretical perspectives.

[13] Jöreskog K., Sörbom D. (1996). LISREL 8: User’s Reference Guide.

[14] Kane R. (1999). Responsibility, Luck, and Chance: Reflections on Free Will and Indeterminism. Journal of Philosophy.

[15] Keller J. (2005). In genes we trust: The biological component of psychological essentialism and its relationship to mechanisms of motivated social cognition. Journal of Personality and Social Psychology.

[16] Kline R. B. (1998). Principles and practices of structural equation modeling.

[17] Krueger F., Hoffman M., Walter H., Grafman J. (2014). An fMRI investigation of the effects of belief in free will on third-party punishment. Social cognitive and affective neuroscience.

[18] Leotti L. A., Iyengar S. S., Ochsner K. N. (2010). Born to choose: The origins and value of the need for control. Trends in cognitive sciences.

[19] Lynn M. T., Muhle-Karbe P. S., Aarts H., Brass M. (2014). Priming determinist beliefs diminishes implicit (but not explicit) components of self-agency. Frontiers in psychology.

[20] Lynn M. T., Van Dessel P., Brass M. (2013). The influence of high-level beliefs on self-regulatory engagement: evidence from thermal pain stimulation.

[21] MacCallum R. C., Widaman K. F., Zhang S., Hong S. (1999). Sample size in factor analysis. Psychological methods.

[22] Nahmias E. (2005). Agency, authorship and illusion. Consciousness and Cognition.

[23] Nahmias E., Morris S. G., Nadelhoffer T., Turner J. (2006). Is incompatibilism intuitive?. Philosophy and Phenomenological Research.

[24] Nichols S., Knobe J. (2007). Moral Responsibility and Determinism: The Cognitive Science of Folk Intuitions. Noûs.

[25] Paulhus D. L., Carey J. M. (2011). The FAD-Plus: measuring lay beliefs regarding free will and related constructs. Journal of Personnality Asessment.

[26] Paulhus D. L., Margesson A. (1994). Free Will and Scientific Determinism (FAD-4) scale.

[27] Pereboom D. (2001). Living without Free Will.

[28] Plaisant O., Courtois R., Réveillière C., Mendelsohn G. A., John O. P. (2010). Validation par analyse factorielle du Big Five Inventory français (BFI-Fr). Analyse convergente avec le NEO-PI-R. Annales Médico-psychologiques, revue psychiatrique.

[29] Rakos R. F., Laurene K. R., Skala S., Slane S. (2008). Belief in free will: Measurement and conceptualization innovations. Behavior and Social Issues.

[30] Rigoni D., Kühn S., Sartori G., Brass M. (2011). Inducing disbelief in free will alters brain correlates of preconscious motor preparation the brain minds whether we believe in free will or not. Psychological science.

[31] Rigoni D., Pourtois G., Brass M. (2015). ‘Why should I care?’ Challenging free will attenuates neural reaction to errors. Social Cognitive and Affective Neuroscience.

[32] Saroglou V., Galand P. (2004). Identities, values, and religion: A study among Muslim, other immigrant, and native Belgian young adults after the 9/11 attacks. Identity: An International Journal of Theory and Research.

[33] Saroglou V., Munoz-Garcia A. (2008). Individual differences in religion and spirituality: An issue of personality traits and/or values. Journal for the Scientific Study of Religion.

[34] Shariff A. F., Greene J. D., Karremans J. C., Luguri J. B., Clark C. J., Schooler J. W., Baumeister R. F., Vohs K. D. (2014). Free Will and Punishment A Mechanistic View of Human Nature Reduces Retribution. Psychological science.

[35] Stillman T. F., Baumeister R. F. (2010). Guilty, free, and wise: Determinism and psychopathy diminish learning from negative emotions. Journal of Experimental Social Psychology.

[36] Stillman T. F., Baumeister R. F., Vohs K. D., Lambert N. M., Fincham F. D., Brewer L. E. (2010). Personal philosophy and personnel achievement: Belief in free will predicts better job performance. Social Psychological and Personality Science.

[37] Strawson G. (1986). Freedom and Belief.

[38] Stroessner S. J., Green C. W. (1990). Effects of belief in free will or determinism on attitudes toward punishment and locus of control. Journal of Social Psychology.

[39] Taylor S. E., Brown J. D. (1988). Illusion and well-being: a social psychological perspective on mental health. Psychological Bulletin.

[40] Viney W., Waldman D., Barchilon J. (1982). Attitudes toward punishment in relation to beliefs in free will and scientific determinism. Human Relations.

[41] Vohs K. D., Schooler J. W. (2008). The value of believing in free will encouraging a belief in determinism increases cheating. Psychological science.

[42] Wegner D. M. (2002). The Illusion of Conscious Will.

