# Supplementary material for: What Do You Believe In? French Translation of the FAD-Plus to Assess Beliefs in Free Will and Determinism and Their Relationship with Religious Practices and Personality Traits
Source: Psychol Belg. 2017 Feb 20;57(1):1–16. doi: 10.5334/pb.321 (PMC5808107; doi:10.5334/pb.321)
Supplement: Supplementary file 1 [file pb-57-1-321-s1.pdf]

APPENDIX

FAD-plus : Free Will and Scientific Determinism

Pour chaque proposition ci-dessous, choisissez un chiffre allant de 1 à 5 afin d'indiquer si vous êtes d'accord ou pas d'accord avec la proposition.

1	2	3	4	5
Tout à fait d'accord				Pas du tout d'accord

1	Je crois que l'avenir est déterminé par le sort	**1 2 3 4 5**
2	La constitution biologique des personnes détermine leurs talents et leur personnalité	**1 2 3 4 5**
3	Les évènements dus au hasard semblent être la cause majeure de l'histoire de l'humanité	**1 2 3 4 5**
4	Les gens ont un contrôle complet sur les décisions qu'ils prennent	**1 2 3 4 5**
5	Quelques soient les efforts que vous faites, vous ne pouvez pas changer votre destin	**1 2 3 4 5**
6*	Les psychologues et les psychiatres finiront par comprendre tout le comportement humain	**1 2 3 4 5**
7	Personne ne peut prédire ce qui va arriver dans ce monde	**1 2 3 4 5**
8	Les gens doivent endosser la pleine responsabilité des mauvais choix qu'ils ont fait	**1 2 3 4 5**
9	Le destin est déjà planifié pour chacun	**1 2 3 4 5**
10	Tes gènes déterminent ton avenir	**1 2 3 4 5**
11	La vie semble imprévisible, comme lancer un dé ou jouer à pile ou face	**1 2 3 4 5**
12	Les gens peuvent surmonter tous les obstacles s'ils en ont vraiment envie	**1 2 3 4 5**
13	Ce qui doit arriver arrivera, il n'y a pas grande chose que tu puisses faire	**1 2 3 4 5**
14*	La science a montré comment ton environnement passé a créé ton intelligence et ta personnalité	**1 2 3 4 5**
15*	Les gens sont imprévisibles	**1 2 3 4 5**
16	Les criminels sont totalement responsables des mauvaises actions qu'ils ont faites	**1 2 3 4 5**
17	Que les gens aiment cela ou pas, des forces mystérieuses influencent leurs vies	**1 2 3 4 5**
18*	Comme pour les autres animaux, le comportement humain suit toujours les lois de la nature	**1 2 3 4 5**
19	La vie est difficile à prédire car elle est presque entièrement aléatoire	**1 2 3 4 5**
20	La chance joue un rôle important dans la vie des personnes	**1 2 3 4 5**
21	Les gens disposent d'un libre-arbitre complet	**1 2 3 4 5**
22	Le caractère des parents détermine celui de leur enfant	**1 2 3 4 5**
23	Les gens sont toujours en tort pour leur mauvais comportement	**1 2 3 4 5**
24	L'environnement que tu as eu pendant ton enfance détermine ton succès en tant qu'adulte	**1 2 3 4 5**
25	Ce qui arrive aux gens est une question de hasard	**1 2 3 4 5**
26*	La force de l'esprit peut toujours surmonter les désirs du corps	**1 2 3 4 5**
27*	L'avenir des gens ne peut pas être prédit	**1 2 3 4 5**

**Items in grey are recommended not to be used in French unless additional reliable analyses*
